# Three-Dimensional Multi-Material Topology Optimization: Applying a New Mapping-Based Projection Function

**DOI:** 10.3390/ma18050997

**Published:** 2025-02-24

**Authors:** Hélio Luiz Simonetti, Francisco de Assis das Neves, Valério Silva Almeida, Marcio Maciel da Silva, Luttgardes de Oliveira Neto

**Affiliations:** 1 Department of Mathematics, Federal Institute of Education, Science and Technology of Minas Gerais (IFMG), Betim 32677-562, Brazil; 2 Department of Civil Engineering, Federal University of Ouro Preto (UFOP), Ouro Preto 35400-000, Brazil; fassis@ufop.edu.br (F.d.A.d.N.); marcio.maciel@aluno.ufop.edu.br (M.M.d.S.); 3 Department of Geotechnical and Structural Engineering, School of Engineering, University of São Paulo (USP), Sao Paulo 13566-590, Brazil; valerio.almeida@pq.cnpq.br; 4 Department of Civil and Environmental Engineering, Sao Paulo State University (UNESP), Bauru 17033-360, Brazil; luttgardes.oliveira-neto@unesp.br

**Keywords:** topology optimization, multi-material, MATLAB code, sigmoid function, 3D structures

## Abstract

This paper presents an efficient and compact MATLAB code for 3D topology optimization of multi-materials. The multi-material problem using a mapping-based material interpolation function is adopted from previous work, in which each material is modeled in the same way, presenting a clear (clean) result of 0 and 1 for each material of the optimized structures, without gray elements, thus facilitating the manufacturing process. A new projection function, the sigmoid function, is adopted for the filtered design variables for each material in the domain. The proposed method improves computational efficiency, reducing computational costs by up to 36.7%, while achieving a 19.1% improvement in the objective function compared to the hyperbolic tangent function. A multi-material topology optimization solution with minimal compliance under volume constraints, including details of the optimization model, filtering, projection, and sensitivity analysis procedures, is presented. Numerical examples are also used to demonstrate the effectiveness of the code, and the influence of the position of the support on the optimized results is also proven. The complete MATLAB code for 3D elastic structures is presented as an example.

## 1. Introduction

Single-material topology optimization (TO) seeks the best distribution of a given material within the design domain so that the resulting structure, among other goals, minimizes compliance, minimizes von Mises stress growth, or maximizes the first natural frequency, subject to a volume constraint to maximize its performance. There are several approaches to defining design variables, such as those based on elements used by the Solid Isotropic Material with Penalization (SIMP) method by [1,2]. The Evolutionary Structural Optimization (ESO) methods by [3,4] and their variants: the Bi-directional Evolutionary Structural Optimization (BESO) method [5,6,7] and the Smooth Evolutionary Structural Optimization (SESO) method by [8,9]. Or even, limit-based approaches, such as the Level Set Method (LSM) by [10,11,12], are also considered.

In the last decade, Multi-Material Topology Optimization (MMTO) has attracted a lot of attention from researchers due to the advent of multi-material additive manufacturing. Bandyopadhyay and [13,14] highlight that multi-material manufacturing can be easily printed from a computer point-by-point and layer-by-layer.

The Level Set Method is widely used in multi-material optimization due to its ability to represent complex geometries implicitly, handle topology changes, and allow for easy incorporation of multiple materials. The classical LSM was extended to MMTO by [15]. For *m* materials, log(2*m*) was used for the level set, and the structure was updated using a set of Hamilton–Jacobi equations. The multi-level set modeling method was extended for stress-constrained multi-material topology optimization by [16,17]. A level-based multi-material topology optimization method using a reaction–diffusion equation was proposed by [18]. The modified multi-material description of the Multi-Material Level Set (MM-LS) was introduced by [19], which also has the advantage that each phase is represented by a combined formulation of different level set functions. A new MMTO strategy based on the Material-Field Series-Expansion (MFSE) model was proposed for a structure composed of *m* different solid material phases [20]. In this approach, *m* individual material fields are introduced to describe the topology distribution in the model’s multi-material representation.

Using the element approach, a SIMP-type multi-material interpolation model was proposed in [21], first interpolating between two non-zero phases and then between ’solid’ (material) and ’void’ (absence of material). An MMTO approach was introduced by dividing the problem into a series of 0–1 TO subproblems [22]. The SIMP model was employed between two adjacent materials, and the multi-material BESO method was proposed in [23]. MMTO was extended under multiple volume and/or mass constraints using the Discrete Material Optimization (DMO) technique [24,25], while material nonlinearity was considered in [26,27]. Multi-material topology optimization, addressing multi-material volumes, has also been applied to diverse problems, including cable-suspended membrane structures, combined structural and thermal analyses, thermal buckling criteria, and lattice structures, as explored in [28,29,30,31], respectively.

One of the bottlenecks of MMTO is the interpolation functions, with the selection of the initial values of the design variables being crucial, as it can significantly influence the optimization result. If the initial values are not well chosen, the optimizer can get stuck in local optima. A mapping-based interpolation function for MMTO is proposed in [32] as an alternative to polynomial interpolation functions or SIMP-based interpolation functions. The proposed function combines the p-norm of the design variables assigned to each finite element and the 1-norm of the design variable. In this sense, this article also contributes a new projection function for the design variables filtered for each material in the domain, implemented through the sigmoid function, which is smoother than the hyperbolic tangent function used by [32]. This results in a clearer transition between the boundaries of each material in the optimized structure.

Despite recent advancements, current multi-material topology optimization (MMTO) methods still face significant challenges, particularly concerning interpolation functions and intermediate-density regions. MMTO formulations often rely on interpolation schemes, such as polynomial or SIMP-based methods, to transition between materials. However, these approaches frequently result in intermediate-density regions, which complicate manufacturability. While some studies have attempted to address this issue using projection functions, many still suffer from abrupt transitions or numerical instabilities. To mitigate these problems, this article introduces a new projection function based on the sigmoid function, which ensures smoother transitions and improved numerical stability.

Another critical challenge is the computational cost and convergence difficulties associated with MMTO. The inclusion of multiple materials increases the number of design variables and constraints, leading to higher computational demands and slower convergence rates. This issue is especially pronounced in large-scale 3D problems, where traditional MMTO methods often struggle to deliver efficient solutions. Although iterative solvers like the Preconditioned Conjugate Gradient (PCG) method are effective for single-objective optimization due to their simplicity and low computational cost, they may not be suitable for more complex problems involving multiple conflicting objectives. In practical applications, multi-objective optimization is often necessary to address real-world engineering constraints. In such cases, alternative strategies, such as Pareto Front Methods (e.g., NSGA-II and MOEA/D) and scalarization techniques (e.g., the ε-constraint method and the weighted sum method), are more appropriate. These methods enable trade-offs between objectives like stiffness, weight, and cost, with the weighted sum method being particularly popular due to its ease of implementation and straightforward formulation.

To address these challenges, this work proposes a sigmoid-based projection function for MMTO, which enhances the transition between material and void regions while significantly reducing computational costs. Unlike the hyperbolic tangent function, the sigmoid function provides a more gradual and stable transition, effectively eliminating intermediate-density elements without introducing abrupt changes that could compromise structural integrity. Numerical results demonstrate that the proposed approach achieves up to a 36.7% reduction in computational cost and a 19.1% improvement in the objective function compared to traditional projection methods, making it a promising alternative for large-scale 3D MMTO problems.

This paper focuses on enhancing projection functions in MMTO to achieve smoother and more efficient transitions between material and void regions within the design domain. While hyperbolic tangent functions have been widely used, they exhibit drawbacks such as abrupt transitions that can lead to undesirable stress concentrations and less refined solutions. The proposed sigmoid function addresses these issues by improving problem discretization, optimizing material distribution, and reducing computational costs. Additionally, it aligns with the requirements of multi-material additive manufacturing, delivering optimized designs with clearly defined features and eliminating intermediate-density elements.

To demonstrate the effectiveness of the proposed approach, the authors apply MMTO using three materials—concrete (red), aluminum (blue), and steel (green)—to a cantilever beam, an MBB beam, and a bridge structure with two types of supports. These examples highlight the influence of boundary conditions and the advantages of the sigmoid projection function. Furthermore, a cantilever beam under axial load is analyzed to showcase the algorithm’s capability to handle up to 10 different materials, achieving an intelligent and optimized design solution.

This article is organized as follows. Section 2 describes the formulation of the MMTO problem, including the design variable matrix and the sigmoid projection function, while discussing the limitations of existing projection functions. Section 3 presents the numerical implementation and the iterative solution using Preconditioned Conjugate Gradients (PCG) and Incomplete Cholesky algorithms. Section 4 provides numerical examples comparing the sigmoid projection function with the hyperbolic tangent function, emphasizing the advantages of the proposed approach. Finally, Section 5 concludes the paper.

## 2. The Formulation of the Multi-Material Topology Optimization Problem

### 2.1. Matrix of Design Variables

The formulation of the Multi-Material Topology Optimization (MMTO) problem using a mapping-based interpolation function consists of finding the optimal distribution of the different materials within the design domain, considering volume or mass constraints. For optimization of a single material, a density $x_{e}$ is assigned, and modeling the interpolation function of the problem is easier. In this case, the design variables can be expressed by the vector $x={\left[\begin{array}{ccc} x_{1} & x_{2} & x_{3} \end{array}\ldots \begin{array}{cc} x_{N-1} & x_{N} \end{array}\right]}^{T}$, where N is the number of elements used in the discretization.

In the case of MMTO, the existence of multiple materials in the design domain makes the problem more complex. The design variables are now obtained by multiplying the vector that contains the types of materials by the vector of design variables, that is, a number of elements of discretization, see [32]. Thus, the MMTO design variable matrix ${\left[x\right]}_{MxN}$, has the rows, *M*, representing the material properties and the columns, *N*, representing the index of the finite elements of the discretization of the design domain; that is, the number of design variables and can be expressed as:

(1) $${\left[x_{i}^{j}\right]}_{MxN}=\left[\begin{array}{ccc} \begin{array}{cc} x_{1}^{1} & x_{2}^{1}\\ x_{1}^{2} & x_{2}^{2}\\ x_{1}^{3} & x_{2}^{3} \end{array} & \cdots & \begin{array}{cc} x_{N-1}^{1} & x_{N}^{1}\\ x_{N-1}^{2} & x_{N}^{2}\\ x_{N-1}^{3} & x_{N}^{3} \end{array}\\ \vdots & \ddots & \vdots \\ \begin{array}{cc} x_{1}^{M-1} & x_{2}^{M-1}\\ x_{1}^{M} & x_{2}^{M} \end{array} & \cdots & \begin{array}{cc} x_{N-1}^{M-1} & x_{N}^{M-1}\\ x_{N-1}^{M} & x_{N}^{M} \end{array} \end{array}\right]\;\;\;\forall \left\{\begin{array}{c} i=1,2,\ldots ,N\\ j=1,2,\ldots ,M \end{array}\right.$$

### 2.2. Formulation of the MMTO

In the search for the maximum stiffness topology via MMTO, it is common to employ the minimization of the average compliance or the maximum von Mises stress as an objective function, while the restriction is imposed on the structural weight, limiting the maximum volume of material allowed. As compliance represents the work performed by applied loads in the structure’s equilibrium state, an alternative approach to maximum stiffness design is to use elastic strain energy as a measure of structural stiffness. Thus, the compliance minimization problem can be reformulated as a problem of minimizing the total elastic strain energy. The formulation of the MMTO problem with multiple design variables assigned to each finite element can be defined as:

(2) $$\begin{array}{ll} \mathrm{Minimize}: & C\left(\tilde{x}\right)=U^{T}(\tilde{x})K(\tilde{x})U(\tilde{x})\\ \mathrm{Subject}\;\mathrm{to}: & K(\tilde{x})U(\tilde{x})=F(\tilde{x})\\ & V{\left(\bar{\tilde{x}}\right)}_{i}\leq V_{i}^{0}\forall i=\left\{1,2,3\ldots ,\;M\right\}\\ & 0\leq x\leq 1 \end{array}$$

where *K*, U, and *F* represent the stiffness matrix, the displacement vector, and the force vector, respectively. *C* is the compliance, $V{\left(\bar{\tilde{x}}\right)}_{i}$ is the volume of each material and $V_{i}^{0}$ is the maximum volume fraction allowed for each material, $\bar{\tilde{x}}$ is the designed density and $\tilde{x}$ is the intermediate density. Then, the stiffness matrix designed for the MMTO must be assembled as:

(3) $$K\left(\bar{\tilde{x}}\right)=\sum_{e=1}^{N}k^{e}\left(\varphi_{i}\left(\bar{\tilde{x}}\right)\right)\forall \;i=\left\{1,2,3,\ldots ,M\right\}$$

Here, $K\left(\bar{\tilde{x}}\right)$ represents the total stiffness matrix of the system, which is obtained by summing the individual stiffness matrices of all elements *e* in the domain, weighted by the material interpolation functions $\varphi_{i}\left(\bar{\tilde{x}}\right)$. For the SIMP method, the material interpolation functions based on those proposed by [33,34] are described as follows:

(4) $$k^{e}=\sum_{i=1}^{M}{\left(\varphi_{i}\right)}^{n}\left(E_{i}-E_{void}\right)+E_{void}$$

In this paper, n is the penalization factor, which is used to guide the convergence of the design variable to 0 or 1. $\varphi_{i}$ is the mapping-based interpolation function proposed by [32] and expressed as:

(5) $$\varphi_{i}=\frac{{\left\|{\bar{\tilde{x}}}^{e}\right\|}_{p-norm}}{{\left\|{\bar{\tilde{x}}}^{e}\right\|}_{1-norm}+\delta }{\bar{\tilde{x}}}_{i}^{e}$$

The term $\delta =10^{-9}$ is a very small value used to avoid the mathematical indeterminacy operation $\frac{0}{0}$. Therefore, the higher the value of δ, the lower the precision in the approximation; this strategy is used to balance precision and robustness. Furthermore, $E_{i}$ is the Young’s modulus for the i-th material and $E_{void}$ represents the Young’s modulus of the void regions.

Furthermore, ${\left\|{\bar{\tilde{x}}}^{e}\right\|}_{p-norm}$ and ${\left\|{\bar{\tilde{x}}}^{e}\right\|}_{1-norm}$ are the p-norm and 1-norm of the projected design variables given by:

(6) $${\left\|{\bar{\tilde{x}}}^{e}\right\|}_{p-norm}=\;\sum_{i=1}^{M}{\left[{\left({\bar{\tilde{x}}}_{i}^{e}\right)}^{p}\right]}^{\frac{1}{p}}$$

(7) $${\left\|{\bar{\tilde{x}}}^{e}\right\|}_{1-norm}=\;\sum_{i=1}^{M}{\bar{\tilde{x}}}_{i}^{e}$$

The interpolation function computes the effective Young’s modulus $k^{e}$ of each element by combining the moduli of different materials weighted by the function $\varphi_{i}$. This enables the optimized structure to exhibit desired mechanical properties, adapting to performance requirements. Additionally, the use of p-norm and 1-norm in the interpolation functions allows $\varphi_{i}$ to accurately represent the relative contribution of each material within an element, helping to avoid indeterminacies and ensuring that the sum of contributions from different materials does not exceed the material’s capacity. To ensure robustness in the calculation of the interpolation function, the parameter δ is introduced, preventing indeterminacies and contributing to the stability of the optimization algorithm, especially in regions where design variables approach zero.

### 2.3. Projection of the Filtered Element

To solve mesh dependency and checkerboard problems, a density filter is used, which is already provided in the 88-line MATLAB code (v.R2021b) of [35] and in this extended article for MMTO. Then, the filtered element is calculated using the expression:

(8) $${\tilde{x}}_{i}^{e}=\frac{\sum_{j\in N_{e}}H_{ej}x_{i}^{j}}{\sum_{j\in N_{e}}H_{ej}}$$

where $N_{e}$ is the neighborhood of an element $x_{i}^{e}$ defined as $N_{e}=\left\{j:dist\left(e,j\right)\leq R\right\},$ with *R* being the filter radius, and $H_{ej}$ being the weighting factor defined as $H_{ej}=\max\;(0,$ $R-dist\left(e,j\right))$.

In the SIMP method, it is common to find intermediate densities during the TO procedure. However, to avoid the presence of elements with intermediate densities in the MMTO optimized structures and guarantee a binary (0–1) solution, the projection function originally proposed by [32,36,37], which is a hyperbolic tangent, has been replaced in this article by a sigmoid function. The sigmoid function is a suitable choice for forcing densities to approach 0 or 1 smoothly, ensuring that only binary values are considered in optimization. This helps to simplify the resulting structures and makes their fabrication more feasible. Thus, the projected density is given by Expression (9):

(9) $${\bar{\tilde{x}}}_{i}^{e}=\frac{sigmoid\left(\eta ,\beta \right)+sigmoid({\tilde{x}}_{i}^{e}-\eta ,\beta )}{sigmoid\left(\eta ,\beta \right)+sigmoid(1-\eta ,\beta )}$$

and the projection function is given by Expression (10):

(10) $$sigmoid\left({\tilde{x}}_{i}^{e},\beta \right)=\frac{\alpha }{\left(1+e^{\left(-\beta {\tilde{x}}_{i}^{e}\right)}\right)}-1$$

The parameter *β* is used to control the slope of the function, that is, the sharpness of the projected filtered variable ${\bar{\tilde{x}}}_{i}^{e}$; the parameter η defines the center of the smooth transition part of the function and is selected as 0.5 as proposed in [32]. The parameter α is used to customize the sigmoid function and aims to approximate the sigmoid values with the hyperbolic tangent values.

**Figure 1:**
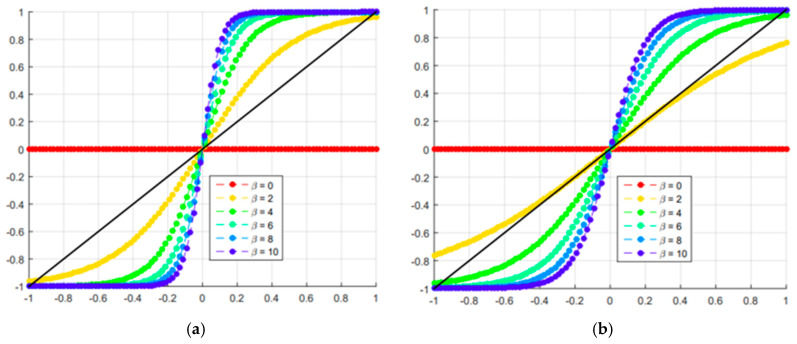
Projection function: (**a**) hyperbolic tangent function; (**b**) sigmoid function.

Note that in the two graphs in Figure 1, the behaviors are similar. However, the sigmoid function is a little smoother than the hyperbolic tangent. It can also be observed that for *β* = 0, the filtered density $\left({\tilde{x}}_{i}^{e}\right)$ is equal to the projected density (${\bar{\tilde{x}}}_{i}^{e});$ that is, the density is filtered linearly; for *β* → ∞ the function acts as a step function. For all other cases of *β*, the function acts as a penalty, forcing the intermediate density to move in the 0 –1 direction.

The use of the sigmoid projection function in multi-material topology optimization leads to better optimal configurations due to its ability to promote smooth transitions between material and void regions, delivering a better distribution of the materials. The hyperbolic tangent, while also providing continuous transitions, tends to exhibit more abrupt changes and discontinuities in the distribution, which can result in less refined solutions and undesirable structural behavior. These discontinuities can lead to unwanted stress concentrations, compromising structural integrity. The sigmoid function, with its mapping restricted between 0 and 1, allows for more delicate control over material configuration, which is essential in problems involving multiple materials. This smoothness in transition is important for avoiding stress concentrations and ensuring a more homogeneous structural response, resulting in configurations that maximize stiffness while minimizing the weight of the structure. Therefore, the sigmoid function proves to be more suitable for seeking efficient solutions in multi-material topology optimization. However, the sigmoid projection function, while effective in smoothing transitions and eliminating intermediate densities, can face challenges in complex geometries where more careful tuning of the β and η parameters is required to balance convergence and accuracy; as smoothing transitions can lead to loss of fine details, potentially impacting structural performance. To address the influence of β in the optimization process, we implemented a dynamic update procedure for β using a linearly increasing function. This procedure allows β to gradually increase every 20 iterations, until reaching the limit of 5.85, which yielded the best topology results. The progressive growth of β ensures a smooth transition at the beginning of the optimization, preventing premature convergence to suboptimal solutions. As β increases, it enforces sharper boundaries between materials, effectively eliminating intermediate densities without compromising numerical stability. The upper limit of 5.85 was empirically determined as the best balance between accuracy, computational efficiency, and convergence behavior across different structural configurations.

### 2.4. Sensitivity Analysis

Sensitivity in structural optimization measures how changes in design variables impact the objective function and constraints. It is critical to understand the effect of these small changes in design variables on the overall performance of the structure. This sensitivity of the objective function C about the design variables $x_{i}^{j}$ can be obtained by the chain rule, and can be calculated as follows:

(11) $$\frac{\partial C}{\partial x_{i}^{j}}=\frac{\partial C}{\partial {\bar{\tilde{x}}}_{i}^{e}\;}\frac{\partial {\bar{\tilde{x}}}_{i}^{e}\;}{\partial {\tilde{x}}_{i}^{e}}\frac{\partial {\tilde{x}}_{i}^{e}}{\partial x_{i}^{j}}$$

Mathematically manipulating Equation (11) and writing the projected variables as a function, we have

(12) $$\frac{\partial C}{\partial {\bar{\tilde{x}}}_{i}^{e}}=-U^{T}\frac{\partial K}{\partial {\bar{\tilde{x}}}_{i}^{e}\;}U$$

The Young’s moduli of each material interpolated by the mapping-based interpolation function can be expressed as follows:

(13) $$E\left({\bar{\tilde{x}}}_{i}^{e}\right)=\sum_{i=1}^{M}\left(\varphi_{i}\right)\left(E_{i}-E_{void}\right)+\;E_{void}$$

Using the quotient rule to derive the interpolation function, Equation (5), about the projected variable and mathematically manipulating Equations (6) and (7) we obtain:

(14) $$\frac{\partial \varphi_{i}}{\partial {\bar{\tilde{x}}}_{i}^{e}}=\;\frac{\left(\sum_{j=1}^{M}{\left[{\left({\bar{\tilde{x}}}_{j}^{e}\right)}^{p}\right]}^{\frac{1}{p}-1}{\left({\bar{\tilde{x}}}_{i}^{e}\right)}^{p}+\sum_{j=1}^{M}{\left[{\left({\bar{\tilde{x}}}_{j}^{e}\right)}^{p}\right]}^{\frac{1}{p}}\right)\left(\sum_{j=1}^{M}{\left({\bar{\tilde{x}}}_{j}^{e}\right)}^{p}+\delta \right)-\left(\sum_{j=1}^{M}{\left[{\left({\bar{\tilde{x}}}_{j}^{e}\right)}^{p}\right]}^{\frac{1}{p}}\right){\bar{\tilde{x}}}_{i}^{e}}{{\left(\sum_{j=1}^{M}{\left({\bar{\tilde{x}}}_{j}^{e}\right)}^{p}+\delta \right)}^{2}}\;$$

## 3. Numerical Implementation

To solve systems of linear equations as defined in the equality constraint, in Equation (2), two types of methods are used: direct and iterative. Since the system is defined by the equation *KU* = *F*, the stiffness matrix *K* is positive definite. Direct solvers generally use Cholesky factorization, which is a variant of Gaussian elimination that takes advantage of the symmetric and positive definite properties to reduce storage, arithmetic, and guarantee stability without pivoting. For positive definite and symmetric linear systems, the Conjugate Gradient (CG) method is one of the most popular approaches in the family of Krylov subspace methods [38]. The convergence of the CG method depends on the condition number of the matrix. The CG method converges faster for well-conditioned matrices.

Typically, the CG method is combined with a preconditioner to accelerate its convergence. The preconditioner is symbolically represented by a matrix; in this article, $K^{-1}$ is used, and the original linear system is replaced by $K^{-1}KU=K^{-1}F$. A good preconditioner should be a good approximation of $K^{-1},$ and solving systems of the type $K^{-1}z=r$ should be relatively cheap. Another requirement is that *K* must be symmetric and positive definite. The CG method minimizes the K-norm of the error in each iteration.

There is extensive literature on preconditioner generation for the CG scheme. Some of the popular preconditioners include incomplete Cholesky factorization, domain decomposition, and block Jacobi. The approximate factorization preconditioners of the CG method are called incomplete Cholesky preconditioners. Early versions of this method limited the preconditioner to having the same sparsity structure as the matrix *K*. Subsequent improvements defined padding levels to allow for more non-zero elements in *K* [39]. This was followed by the notion of incomplete Cholesky preconditioners based on elimination tolerance, where filling is allowed at arbitrary locations as long as the elements exceed a specified tolerance. In Section 3.1, the Preconditioned Conjugate Gradient (PCG) methods employed in our MMTO program are present. Two types of preconditioners, available as MATLAB routines, were implemented to enhance the efficiency and convergence of the PCG method: one based on the diagonal matrix and the other utilizing the Incomplete Cholesky Decomposition.

The diagonal preconditioner is straightforward and computationally inexpensive, providing a basic level of improvement in convergence rates. On the other hand, the Incomplete Cholesky Decomposition preconditioner offers a more sophisticated approach by approximating the Cholesky decomposition of the system matrix.

### 3.1. Iterative Solution: Preconditioned Conjugated Gradients

In the multi-material OT program, the PCG algorithm from MATLAB code is used to solve the systems defined by the equilibrium equation.

%ITERATIVE SOLVER—PRECONDITIONED CONJUGATED GRADIENT % tolit = 10^(-8); maxit = 8000; M = diag(diag(K(freedofs,freedofs))); U(freedofs,:) = pcg(K(freedofs,freedofs),F(freedofs,:),tolit,maxit,M);

This MATLAB code pcg function is used to solve the *KU* = *F* system. The function arguments are described as follows: *K* is the stiffness matrix; *U* is the displacement vector found when solving the system; *F* is the vector of external forces applied to the structure; *tol* is the tolerance that determines when the iterative method should stop. The algorithm continues with the iterative process until the residue is less than or equal to *tol*; *maxit* is the maximum number of iterations allowed for the method; *M* creates a diagonal matrix *M* from the diagonal elements of the matrix *K*, restricted to the rows and columns specified by freedofs.

### 3.2. Incomplete Cholesky

Another precondition used for multi-material TO solve the linear system defined by the equilibrium equation was the incomplete Cholesky from the MATLAB code. The *‘ichol’* method in MATLAB is used to calculate an incomplete Cholesky decomposition of a symmetric, positive definite matrix, in this case, the stiffness matrix *K*. The Cholesky decomposition is a form of matrix factorization that writes the matrix *K* as the product of a lower triangular matrix *L* and its conjugate transpose, *L’*.
%%%%%%%%ITERATIVE SOLVE—INCOMPLETE CHOLESKY %%%%%%%% L = ichol(K(freedofs,freedofs)); U(freedofs,:) = pcg(K(freedofs,freedofs),F(freedofs,:),10^(-8),1000,L,L’);

In MATLAB, *ichol* computes an incomplete approximation of the matrix *L*. This means that some off-diagonal elements of *L* can be approximated as zero to save time and space. This approach is useful in problems where the matrix A is sparse, which is common in many engineering applications. The PCG call arguments in this routine are described as follows: *L* is the lower triangular matrix of the incomplete Cholesky decomposition. *L*’ is the upper triangular matrix, which is the conjugate transpose of *L.*

## 4. Numerical Results

The following examples of structural engineering focus on TO based on minimizing compliance. The geometry and boundary conditions for numerical applications are represented in each case. All numerical examples were processed on a Core i7-2370, eighth Gen notebook, 2.8 GHz CPU with 20.0 GB (RAM). The first two examples presented aim to validate the sigmoid function as a projection function. Therefore, only PCG was used in these examples as a preconditioner for the linear system. In the bridge examples where the influence of boundary conditions (position of supports) on the optimal configuration was analyzed, the Incomplete Cholesky preconditioner was also applied. The Method of Moving Asymptotes (MMA) [40] was employed to solve the multi-material topology optimization (MMTO) problem and update the design variables. The computational setup for these simulations is summarized in Table 1.

### 4.1. Example 1—Cantilever Beam

**Figure 2:**
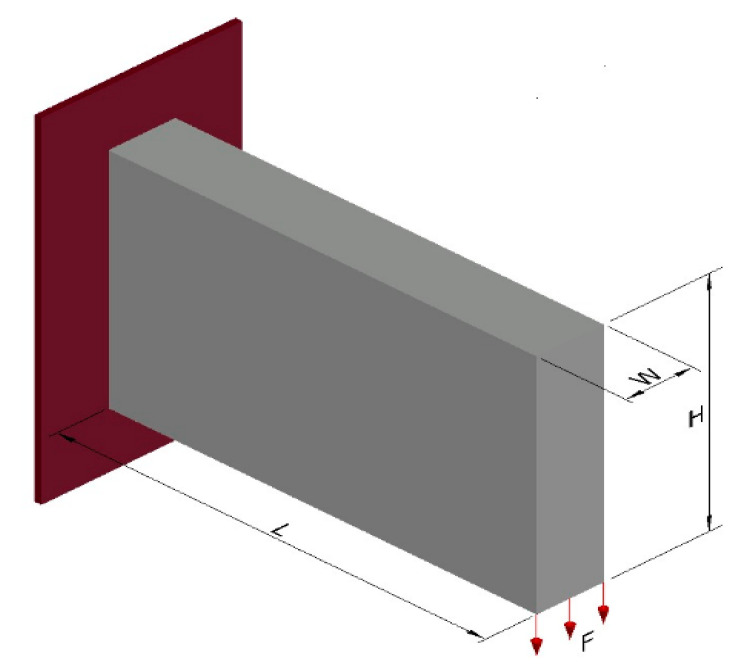
Design domain and boundary conditions.

The cantilever beam shown in Figure 2 is taken as an example of 3D topological optimization with three different types of materials: material 1—concrete with Young’s modulus equal to E(1) = 50 GPa, material 2—aluminum equal to E(2) = 70 GPa, and material 3—steel equal to E(3) = 210 GPa, Poisson’s ratio µ = 0.3, and aims to validate the formulation presented in this article. The design domain and boundary conditions are shown in Figure 2. The left end is fixed and a distributed load of F = 1 kN is applied to the lower right edge. The optimization parameters used are: volume fraction is prescribed at $V_{f}$ = 0.17, filter radius of R = 1.5 m, penalty factor equal to penal = 3, P-norm parameter p = 6. The design domain is discretized with dimensions L = 64 m, H = 40 m, and W = 10 m, totaling 25,600 hexahedral finite elements with an edge length of 1 m. The parameter for the projection function (sigmoid) starts with β = 2.85 and is progressively updated until reaching beta β = 5.85 throughout the iterative process, except for the example with 10 materials, where only β = 5.85 was used. This approach differs from the procedure proposed by [32]. The volume constraints for three materials are set to one-third for each material. This section may be divided by subheadings. It should provide a concise and precise description of the experimental results, their interpretation, as well as the experimental conclusions that can be drawn.

**Figure 3:**
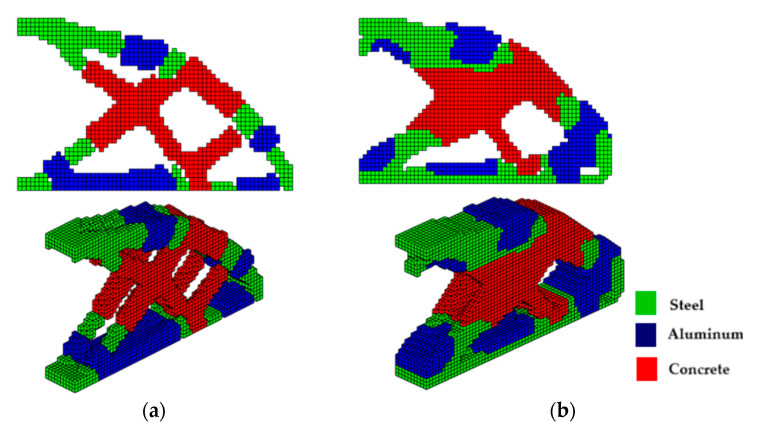
Optimal topologies for three materials: (**a**) Topology using sigmoid function approach of this article; (**b**) Topology using hyperbolic tangent function proposed by [32].

The optimized structure with three materials shown in Figure 3 has a lower compliance than the structure with one material. This is a characteristic of three-dimensional structures with more than one material as these materials increase the rigidity of the structure, reducing the compliance value. Furthermore, the computational cost using the sigmoid for projection was 36.7 min, while the hyperbolic tangent produced a cost of 55.7 min for this topology optimization procedure. It is also noteworthy that the structure via the sigmoid projection function has more defined and thinner regions, in parallel pairs, for all materials, and has the steel region (red) better distributed than the hyperbolic tangent function, which presents the worst distribution of the materials, with massive regions that are denser on the thickness.

### 4.2. Example 2—MBB Beam

**Figure 4:**
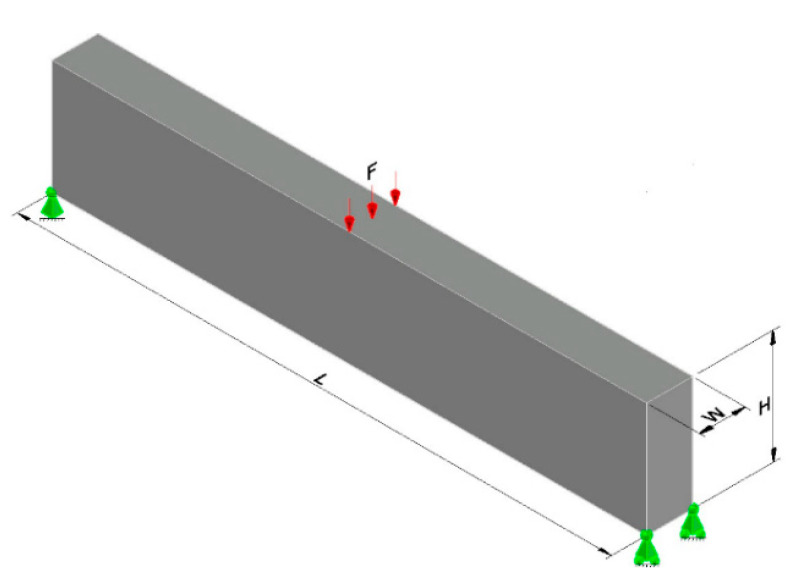
Design domain and boundary conditions.

Figure 4 displays the design domain and boundary conditions for an MBB beam. The parameters used in the multi-material optimization are the same as in example 1. However, for this example, the filter radius was set to *R* = 2.5, and a structured mesh with dimensions *L* = 120 m, *H* = 20 m, and *W* = 4 m was used, resulting in a total of 9600 cubic finite elements, as reported in [41].

**Figure 5:**
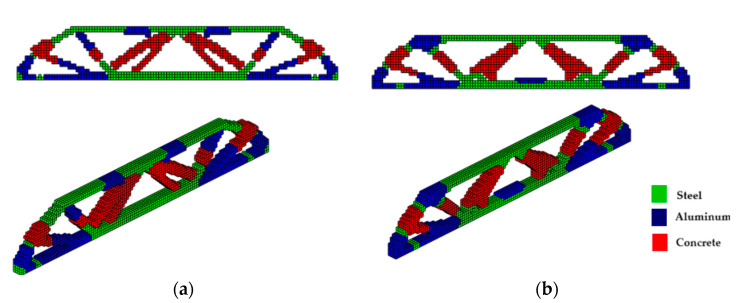
Optimal topologies for three materials: (**a**) Topology using sigmoid function approach of this article; (**b**) Topology using hyperbolic tangent function proposed by [32].

The optimal configurations obtained are shown in Figure 5 and have good accuracy compared to the optimal configurations obtained by other optimization methods with just one material. However, it is observed that the compliance of multi-material optimization is lower in most cases in the literature that presents the values of the objective function. This figure shows that the formulation proposed in this article, together with the formulation by [32], is efficient for three-dimensional elastic structures with multi-materials.

In the MBB beam, only the PCG was used to display the optimal topologies. It was found that both the computational cost and the value of the objective function, using the sigmoid function, present lower values of 32.0% and 4.7%, respectively, in comparison with the hyperbolic tangent function. It is also noted that the optimal topologies presented by the sigmoid function as a projection function exhibit a different material distribution than that proposed by the hyperbolic tangent function. In the upper part, the steel (green) shares space with the aluminum (blue); there is a better definition of regions and slender elements for the sigmoid function, while the hyperbolic tangent function presents less slenderness. The concrete material is positioned in an equal way in both cases.

### 4.3. Example 3—Bridge Topology: Influence of Boundary Conditions

**Figure 6:**
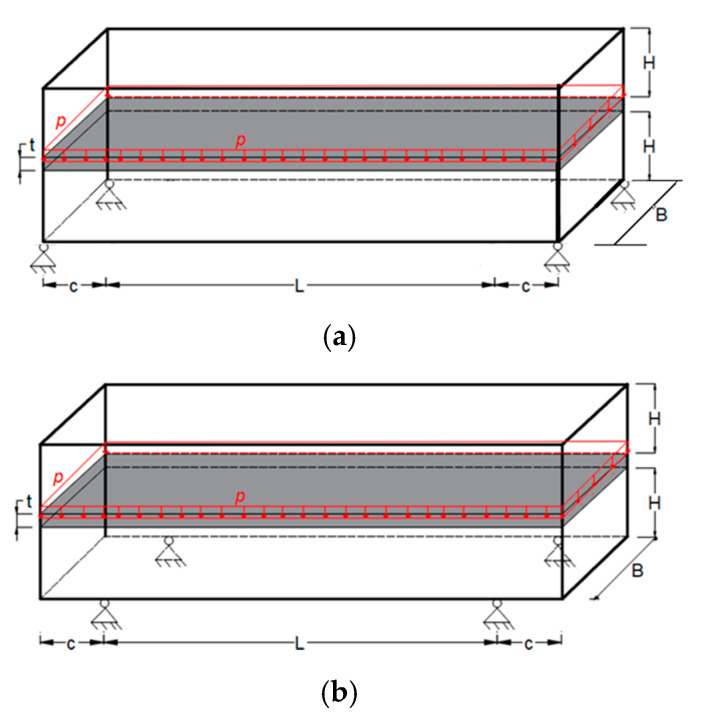
Design domain and boundary condition: (**a**) bridge with end supports (**b**) bridge with recessed supports.

Figure 6 shows the design domain and boundary conditions of a bridge with a central deck. To analyze the influence of boundary conditions on the optimal configuration, the supports were placed at the ends, see Figure 6a, and set back as shown in Figure 6b. In each case, the mesh is defined as 120 × 20 × 10, totaling 24.000 hexahedral finite elements with eight nodes according to [41]. The deck with a thickness of t = 1.0 m, is defined as a non-design domain area, shown as the darker area in Figure 6, with dimensions H = 14.5 m, L = 90 m, c = 15 m, and B = 10.0 m. In this way, the highlighted region will not be allowed to remove solid elements. This section may be divided by subheadings. It should provide a concise and precise description of the experimental results, their interpretation, as well as the experimental conclusions that can be drawn.

**Figure 7:**
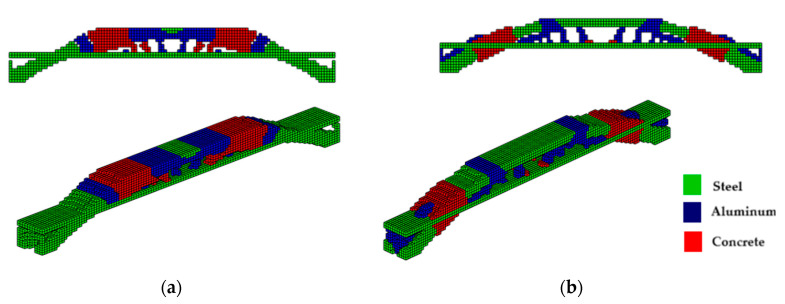
Optimal topologies for three materials with solutions via PCG: supports at the ends—(**a**) Topology using hyperbolic tangent function proposed by [32] and (**b**) Topology using sigmoid function approach of this article.

**Figure 8:**
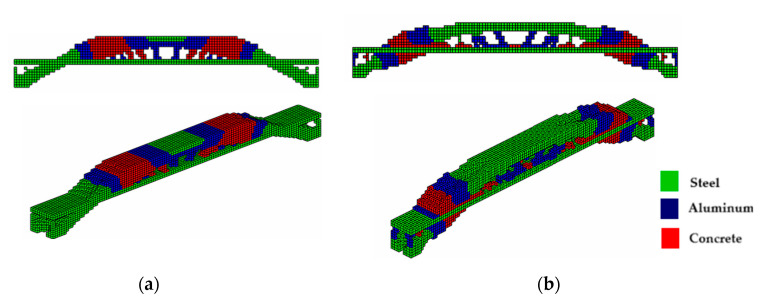
Optimal topologies for three materials with solutions via incomplete Cholesky: bridge with ends supports—(**a**) Topology using hyperbolic tangent function proposed by [32] and (**b**) Topology using sigmoid function approach of this article.

To analyze the formulations presented in this article and compare them with the one presented by [32], the bridges shown in Figure 6 are taken as an example of 3D topological optimization with three materials, and subjected to a distributed load over the central deck. Therefore, the topologies are presented with two numerical formulations to solve the system, see Equation (2), which represents the structure’s equilibrium equation: (1) Preconditioned Conjugate Gradient (PCG), see Figure 7, and (2) Incomplete Cholesky, see Figure 8, considering the supports at the ends of the bridge.

The topologies presented in Figure 7a,b show different geometries for the same optimization parameters. It is worth mentioning that the parameter *β* (Equations (9) and (10)) initially used to solve the system via PCG and Cholesky in both cases is *β* = 2. However, for the hyperbolic function, the parameter *β* doubles every 50 iterations, and for the sigmoid function, it doubles every 40 iterations. The slower change in *β* for the hyperbolic function aims to prevent the system’s stiffness matrix from becoming singular since values of *β* = 2 produce values of the order of magnitude equal to $10^{-18}$. It is also noteworthy that the optimal configurations reached by the PCG with the sigmoid function present an upper geometry of the structure in an arc shape, as expected for a uniformly distributed load, while the hyperbolic tangent function presents an upper trapezoidal geometry. Furthermore, the sigmoid function provides a slenderer geometry and elements than the hyperbolic tangent function.

The optimal topologies illustrated in Figure 8a,b are different in geometry and the distribution of materials involved during the optimization procedure. When the function used is the sigmoid, see Figure 8b; again, an arc can be seen in the upper part with a regular distribution of elements, more steel (green) material, and slender elements. Meanwhile, in Figure 8a, a trapezoid-shaped geometry is seen with a smaller distribution of steel material, with more diagonal cables and thick corners. The sigmoid and the hyperbolic tangent functions are used to avoid the intermediate-density element, that occurs in the material interpolation process, thus guaranteeing a 0–1 solution. It is highlighted here that optimization using the hyperbolic tangent as a projection function has a computational cost 2.3% higher than the sigmoid function, while both yielded the same objective function value.

**Figure 9:**
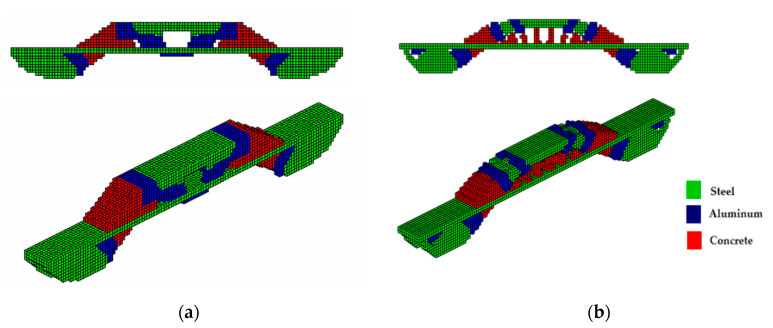
Optimal topologies for three materials with solutions via PCG: with recessed support—(**a**) Topology using hyperbolic tangent function proposed by [32] and (**b**) Topology using sigmoid function approach of this article.

For the bridge shown in Figure 6b, the optimization parameters were kept the same and the optimal topologies are illustrated in Figure 9a,b, using PCG. It is noted that bridges designed with displaced supports at the ends tend to be denser (more material) at these supports due to the distribution of loads. When a vehicle load passes over the bridge deck, the loads are transferred through the bridge structure, referred to as the ‘path of loads’, and discharged onto the supports. The structure engineers aim to ensure the stability and safety of the structure by design analysis. The connection between the end of the bridge and the supports is also important to ensure structural stability and helps to distribute loads more evenly throughout the bridge structure, which is also important to avoid excessive deformations.

**Figure 10:**
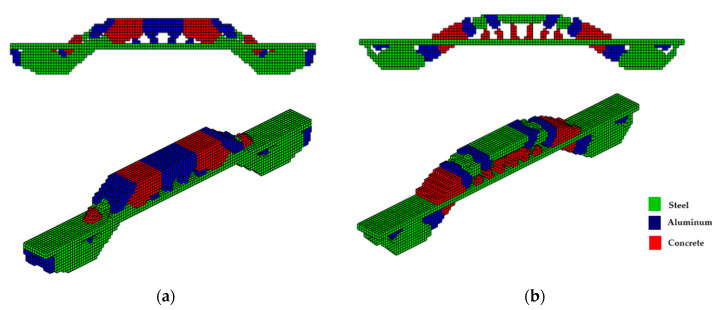
Optimal topologies for three materials with solutions via incomplete Cholesky: with recessed support—(**a**) Topology using hyperbolic tangent function proposed by [32] and (**b**) Topology using sigmoid function approach of this article.

The optimal configurations shown in Figure 10a,b use the incomplete Cholesky preconditioner and are similar to those shown in Figure 9a,b. It is worth highlighting that the computational costs are 1641 s and 1824 s, respectively, for the sigmoid projection and hyperbolic tangent functions. It can also be seen that the use of sigmoid functions provided a 19.1% reduction in the objective function with respect to hyperbolic tangent. In general, in the examples presented in this article, with the two preconditioners applied, the sigmoid function produces a more regular distribution of elements and slenderer structures, even at a lower computational cost than using the hyperbolic tangent function.

### 4.4. Example 4—Cantilever Beam: With Axial Load

**Figure 11:**
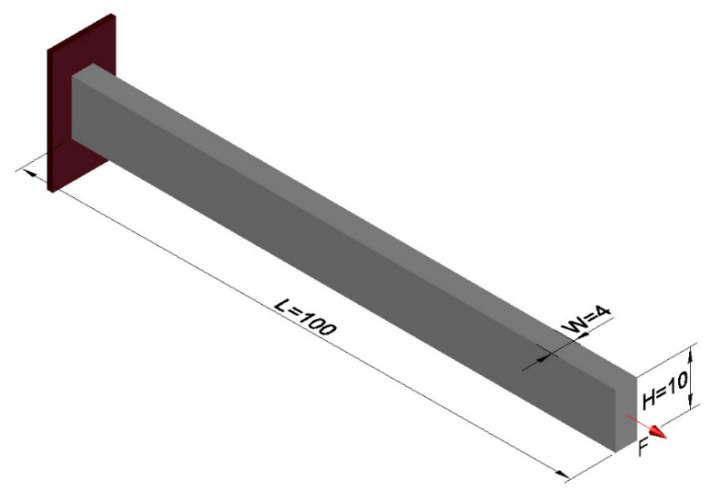
Design domain and boundary conditions.

The formulation of the optimal topology for a structural optimization problem with multiple materials was conducted, considering 10 distinct materials, each with elastic moduli corresponding to *E* = [10,9,8,7,6,5,4,3,2,1]. This approach allowed for the interpolation of mechanical properties, resulting in the precise distribution of materials within the structure’s domain. To demonstrate the effectiveness of the program, a study was conducted on a cantilever beam subjected to an axial load, using the sigmoid function as the optimization method. The main objective was to highlight the algorithm’s ability to accommodate the 10 materials, achieving an intelligent solution for the project. The design domain and boundary conditions are presented in Figure 11, where the domain was discretized into a 100 × 10 × 4 mesh, totaling 4000 eight-node hexahedral finite elements.

**Figure 12:**
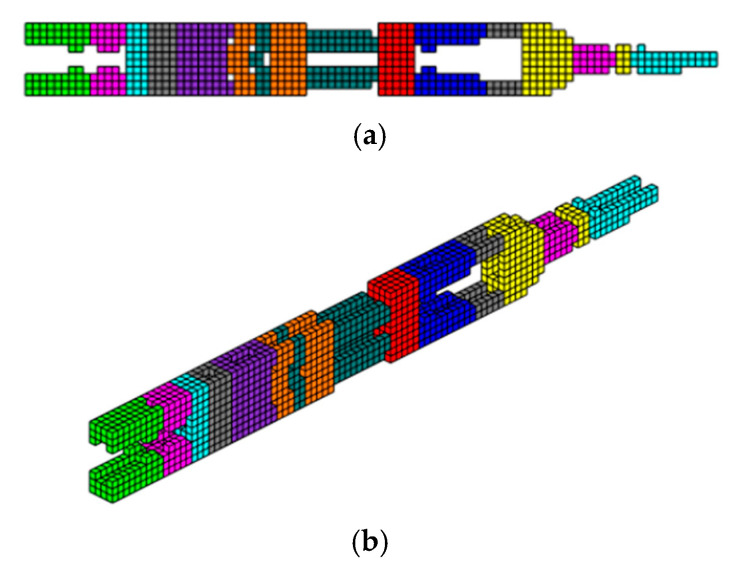
Optimal topologies using 10 materials in increasing order of E: (**a**) Lateral view and (**b**) Oblique view.

In this tensioned cantilever proposed by [24], ten volume constraints are imposed, limiting each material to occupy no more than 5.05% of the total volume of the domain, as shown in Figure 12. Thus, if all materials are used up to this limit, the solution domain will be filled with 50.5% of its volume. This is a feasible design, where the sigmoid projection function mapped the local effects of boundary conditions, distributing the stiffer materials to regions with loading and support conditions.

**Figure 13:**
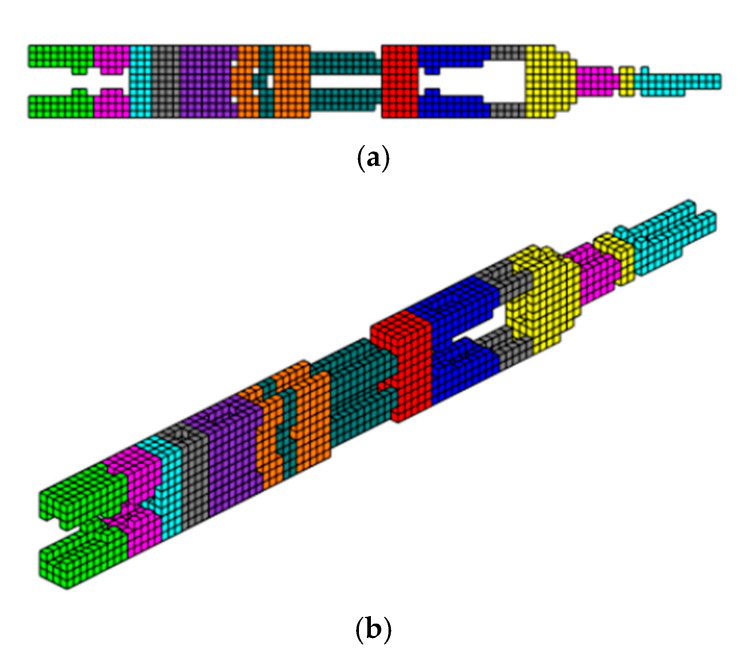
Optimal topologies using 10 materials in decreasing order of E: (**a**) Lateral view and (**b**) Oblique view.

Aiming to show that the permutation of the elements of E does not change the optimal configuration when the projection function used is the sigmoid. The axially loaded cantilever was studied for different permutations with the same optimization parameters and results are shown in Figure 13.

## 5. Discussion

This article addresses one of the key challenges in multi-material topology optimization (MMTO): the formulation of projection functions that ensure smoother and more efficient material distributions, particularly in complex three-dimensional structures. The gap in the literature that this study seeks to fill relates to the need for projection techniques that balance computational efficiency with optimized solutions that eliminate intermediate-density elements. While projection functions such as the hyperbolic tangent have been widely employed, their implementation can result in abrupt discontinuities, leading to undesirable stress concentrations and suboptimal structural performance.

The results highlight the strengths of the proposed sigmoid function. In addition to reducing computational costs by up to 36.7% compared to the hyperbolic tangent, the method achieves a better distribution of materials within the domain, especially in applications involving multiple materials. The sigmoid function was observed to promote smoother transitions between material (solid) and void regions, which not only improves structural homogeneity but also meets the growing demands of multi-material additive manufacturing. This advancement is particularly relevant given the increasing interest in applying combined materials to optimize structural performance.

However, some limitations of the study warrant attention. Firstly, the approach was tested on a limited set of representative examples, such as cantilever beams and bridge configurations. While the results are promising, it would be beneficial to extend the analysis to industrial-scale problems or more complex topologies, such as structures with geometric nonlinearity. Furthermore, the influence of different model parameters, such as the values of β in the sigmoid projection function, could be more comprehensively explored, including sensitivity analyses to better understand the impact of these choices on the final results.

Finally, this article demonstrates the effectiveness of combining innovative projection functions with efficient iterative methods, such as the Preconditioned Conjugate Gradient (PCG) and Incomplete Cholesky Decomposition, to address multi-material optimization problems robustly and computationally feasibly. In this aspect, the work shows in the examples presented that the two preconditioners applied allow the sigmoid function to produce a more regular distribution of elements and slenderer structures, even a lower computational cost than using the hyperbolic tangent function.

Future research could explore the application of this approach to other optimization criteria, such as controlling maximum displacements or minimizing localized stresses, as well as evaluate the impact of emerging manufacturing technologies on its practical implementation.

The results of the sensitivity analysis showed that lower values of η (e.g., 0.25) result in less smoothing of the transition between materials, reducing the computational cost to 442 s, while higher values (0.50 and 0.75) promote a better distribution of materials, but with an increase in the computational cost, respectively, to 595 and 772 s. Among these, η = 0.50 presented the highest computational cost, as it balances the smoothing of the transition and the definition of the material boundaries, resulting in a more uniform and structured distribution. With η = 0.75, it was observed that the concrete tends to concentrate more densely in a single region, reducing the dispersion of materials in the domain, see Table 2.

**Figure 14:**
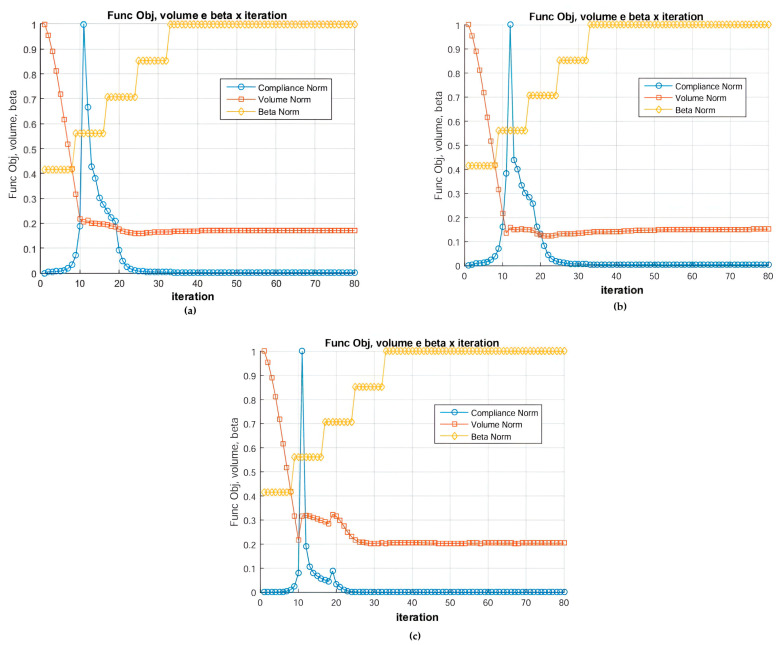
Graph of objective function, volume, and beta by iteration.

Figure 14 shows the graph of the objective function (compliance), volume and beta by the number of iterations. It is noted that the variation in β in the interval [2.85, 5.85] showed that higher values effectively eliminate intermediate densities, making the boundaries between materials more defined and demonstrating how these parameters influence the convergence and distribution of materials, validating the choice of η = 0.50 as the best alternative between topology quality and computational efficiency.

## 6. Conclusions

In this work, MMTO is used to design structures with optimal material distributions, considering multiple materials and volume or mass constraints. It discusses numerical implementation using iterative methods such as Preconditioned Conjugate Gradients (PCG) and Incomplete Cholesky, in addition to analyzing the influence on optimized solutions of adopting the sigmoid projection function suggested by the authors with the hyperbolic tangent function from previous work. Thus, it is possible to conclude.

(1) The sigmoid projection function efficiently smooths the transition between density 0–1, making the problem discrete.
(2) The numerical examples show that the use of the sigmoid function as a projection function shows promising results, with optimized topologies that present an efficient and regular distribution of material and lower computational cost compared to the hyperbolic tangent function.
(3) The use of preconditioners accelerates the convergence of the multiple materials optimization problem procedure and reduces computational cost and processing memory.
(4) It is noteworthy that changing the order of material values $E_{i}$ in the mapping-based material interpolation function does not influence the optimized solution (material distribution), regardless of the order in which the material values $E_{i}$ are presented. This is not the case with the SIMP method of material interpolation.
(5) The current implementation focuses on linear elastic materials under static loads; the methodology can be extended to handle nonlinear and dynamic effects, provided that appropriate numerical schemes are incorporated. This would be an interesting direction for future research.

Therefore, it is highlighted that the approach presented in this article contributes to advances in MMTO, providing more efficient and viable solutions for structural engineering projects.

## Figures and Tables

**Table 1:** Material Properties and Associated Colors (Examples 1 to 3).

Material	Modulus of Elasticity—E	Color
Steel	210 GPa	Green
Aluminum	70 GPa	Blue
Concrete	50 GPa	Red

**Table 2:** Influence of the parameters of the sigmoid function on TO.

β	η	Aluminum	Concrete	Steel	Optimal Topology
[2.85,5.85]	0.25	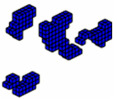	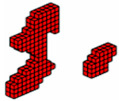	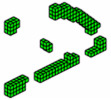	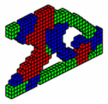
[2.85,5.85]	0.5	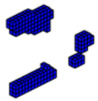	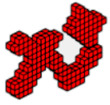	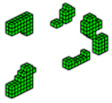	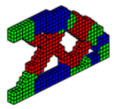
[2.85,5.85]	0.75	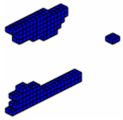	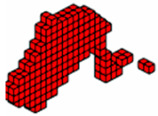	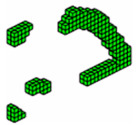	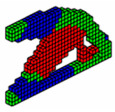

## Data Availability

The original contributions presented in this study are included in the article. Further inquiries can be directed to the corresponding author.
